# A Remedy for Springing of Plates

**Published:** 1856-06

**Authors:** Augustus B. Smith


### A REMEDY FOR SPRINGING OF PLATES.

BY AUGUSTUS B. SMITH.

I have noticed that there are many articles written upon the springing of plates, in the different dental works, and it may appear to some to be but presumption to offer anything new upon the subject, unless it is something that is simple, convenient, and perfect. I will not claim mine to be a sovereign remedy, but leave others to practice and judge for themselves. My plan is the following:

When the case is ready to imbed in the mortar, to line and solder, I form my frame work to it in such a manner that when it is encased, the frame will only be one-eighth of an inch from the teeth. The frame work is made in this manner: Take three pieces of annealed iron wire, say about No. 16, and five or six inches in length; lay these side by side, and twist or braid the ends together, so that the wires or strands in the centre may be spread open and remain in the position you desire them. This is bent to fit around the anterior side of the teeth, one strand to be near the cutting edge, one the centre, and the third at the base or fang of the teeth. One frame of this kind may last to solder fifty cases, by simply bending with the fingers to suit the size of each case. When all is ready, I heap the mortar upon a small thin board kept for this purpose; place the case upon it, press it down, or jar the board which will settle it gradually, until the mixture between the case and board is very thin, or till the plate at the heel either touches the board, or nearly so. I then draw the frame as near the teeth as possible without touching them. When the envelop is sufficiently set, I trim off the superfluous plaster, till the wires become exposed. This you perceive leaves but a very thin covering around the teeth. With a thin knife-blade, or what is still better, as not being so liable to scratch the plate, one made of stiff sheet brass or German silver, I divide the plaster into two equal parts, commencing between the central incisors at their cutting edge, passing over the alveolar ridge and following the median line back to the palatal edge of the plate, cutting carefully down to the plate or nearly so. After the teeth are lined, and ready to heat up, I pass a single wire across the heel of the case, and fasten to the ends of the frame. This gives additional strength, and sustains more surely the plaster in its proper position during the soldering.

My reasons for adopting this plan are few and simple:

1st.—Plaster of Paris heated up to the degree of heat sufficient to solder a case, will shrink about a quarter of an inch to every three inches. It is easy to understand that this has a great tendency to take with it, as far as possible, so yielding a substance as a red hot plate.

2d.—The more of an unshrinkable substance that can be used in connection with the plaster, and still be strong enough for the purpose, reduces the difficulty. Every dentist finds by practice, that half sand and half plaster by measure is that proportion, but this does not fully obviate it. By dividing it in the manner spoken of, reduces it altogether or nearly so. Each side acting independently of the other, cannot, as if it were whole, draw itself inwardly and warp the plate.

3d.—Having so slight a covering around the teeth, makes it easier to solder, the heat not being conveyed away so rapidly as if more substance were used, and of course the less body, the less will be the shrinkage. I believe the great difficulty in springing of plates lies principally in the plaster instead of the metal.—*New York Dental Recorder.*

